# Book Notices and Pamphlets Received

**Published:** 1877-08-20

**Authors:** 


### BOOK NOTICES AND PAMPHLETS RECEIVED.

The Practical Reference Book—Adapted to the use of the Physician, the Pharmacist and the Student. By Richard J. Dunglison, M.D., Philadelphia. Lindsay & Blakiston, Philadelphia.

This is a work of 335 pages, ably gotten up and neatly published; furnishing, in a compact form, information most important and desirable upon subjects like the following: metrical weights and measures; doses of medicine, including recent therapeutical agents; subcutaneous injections, incompatibles, modern treatment of diseases, selected prescriptions, examination of urinary deposits, dietetic preparations, post mortem examinations, etc., etc. The facts and hints collected in this volume are such as every physician should know, and many of which he could not learn except from a vast amount of reading, and from numerous professional treatises.

Diseases of the Sternum, by violent action of the diaphragm during coughing. By F. J. Lutz, A.M., M.D. An interesting paper read before the St. Louis Medical Socieiy

Analysis of Seven Hundred and Seventy-Four Cases of Skin Diseases, treated at the Demilt Dispensary during the year 1876, with remarks on treatment. By L. Duncan Bulkley, A.M., M.D., Physician to the Skin Department Demilt Dispensary, New York, Fellow of the New York Academy of Medicine, etc., etc. D. Appleton & Co.

A valuable paper upon a class of diseases too little studied and understood by the profession.

A Paper on Surgery of the Hand. By E. T. Easley, A.M., M.D., Little Rock, Ark.

A pamphlet of 28 pages, well written, and containing valuable practical suggestions, well defined in the initial paragraph as follows: "1. Some account of the anatomy of the part; 2. The mortality from operations and injuries; 3. Conservatism as applied to its surgery; and, 4. Suggestions as to operative proceedings."

